# Discovery of Ternary Silicon Titanium Nitride with Spinel-Type Structure

**DOI:** 10.1038/s41598-020-64101-5

**Published:** 2020-04-30

**Authors:** Shrikant Bhat, Abhijeet Lale, Samuel Bernard, Wei Zhang, Ryo Ishikawa, Shariq Haseen, Peter Kroll, Leonore Wiehl, Robert Farla, Tomoo Katsura, Yuichi Ikuhara, Ralf Riedel

**Affiliations:** 1 Photon Science, Deutsches Elektronen-Synchrotron DESY, D-22607 Hamburg, Germany; 2 University of Limoges, CNRS, IRCER UMR 7315, F-87000 Limoges, France; 3 Institute of Engineering Innovation, University of Tokyo, Bunkyo, Tokyo, 113-8656 Japan; 4 Japan Science and Technology Agency, PRESTO, Kawaguchi, Saitama, 332-0012 Japan; 5 Department of Chemistry and Biochemistry, University of Texas at Arlington, Arlington, Texas 76019 United States; 6 Bayerisches Geoinstitut (BGI), University of Bayreuth, 95440 Bayreuth, Germany; 7 Fachbereich Material- und Geowissenschaften, Fachgebiet Disperse Feststoffe, Technische Universität Darmstadt, Otto-Berndt-Str. 3, D-64287 Darmstadt, Germany

**Keywords:** Materials science, Condensed-matter physics, Solid-state chemistry, Materials chemistry

## Abstract

Here we report on the discovery of a ternary silicon titanium nitride with the general composition (Si_1−x_,Ti_x_)_3_N_4_ with x = 0 < x < 1 and spinel-type crystal structure. The novel nitride is formed from an amorphous silicon titanium nitride (SiTiN) precursor under high-pressure/high-temperature conditions in a large volume high-pressure device. Under the conditions of 15–20 GPa and 1800–2000 °C, spinel-type γ-Si_3_N_4_ and rock salt-type c-TiN are formed. In addition, crystals of the discovered nano-sized ternary phase (Si_1−x_,Ti_x_)_3_N_4_ embedded in γ-Si_3_N_4_ are identified. The ternary compound is formed due to kinetically-controlled synthesis conditions and is analyzed to exhibit the spinel-type structure with ca. 8 atom% of Ti. The Ti atoms occur in both Ti^3+^ and Ti^4+^ oxidation states and are located on the Si sites. The ternary nano-crystals have to be described as (Si,Ti)_3_N_4_ with N-vacancies resulting in the general composition (Si^4+^_1−x_ Ti^4+^_x-δ_Ti^3+^_δ_)_3_N_4-δ_.

## Introduction

**Figure 1:**
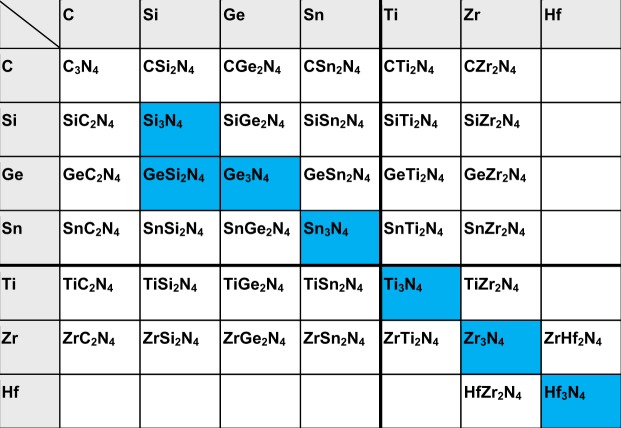
Overview of some high-pressure nitrides experimentally synthesized and theoretically predicted, after W.-Y. Ching *et al*.^4^.

Spinel-type silicon nitride γ-Si_3_N_4_ is a high-pressure phase of silicon nitride and has been discovered in 1999^1^. In the same year, from the group 14 element, spinel type Ge_3_N_4_ ^2^ and Sn_3_N_4_ ^3^ have been found afterwards. Based on these experimental findings, Ching *et al*. predicted a variety of binary and ternary nitrides including nitrides of transition metal elements^4^. In Fig. 1, an updated selection of prominent high-pressure nitrides experimentally accomplished (blues background) and those theoretically predicted (white background) but not achieved yet are listed.

According to their calculations, 39 spinel-type nitrides are characterized by interesting functional and structural features ranging from super-hardness via semiconducting to metallic properties^5^. Later on, it has been found experimentally, that the transition metal high-pressure nitrides of M = Ti, Zr and Hf with the stoichiometry M_3_N_4_ do not show the spinel-type structure as forecasted, but appear in the cubic Th_3_P_4_ structure-type^6^. Experimental studies verified that spinel nitride γ-Si_3_N_4_ in particular, but also the transition metal nitrides M_3_N_4_ with M = Zr, Hf combine ultra-high hardness with high thermal stability against decomposition in different environments. This suggests their promising use in potential applications such as cutting tools^7–9^. Due to their predicted semiconducting behaviour, calculations showed that spinel-type nitrides exhibit interesting optoelectronic properties, which may lead to applications such as light-emitting diodes^4,10^.

Figure 1 impressively reveals that the majority of the predicted ternary nitrides have not been synthesized yet. To the best of our knowledge, the spinel-type GeSi_2_N_4_ is the solitary ternary nitride successfully synthesized^11,12^. Other ternary nitrides remained unknown yet though projected in various studies. There are even more theoretical studies predicting the existence of ternary nitrides e.g. with perovskite-type-based structures^13^ and others^14^ which could further immensely extend the field of unknown nitrides. Recently, a few new ternary nitrides with different structure types were synthesized by Sun *et al*.^15,16^ based on a calculated large stability map of the inorganic ternary metal nitrides. They reported thin films of Zn-based and Mg-based ternary nitrides with wurtzite-type and rock salt-type structures, respectively^16^.

The forecasted ternary nitrides open up further numerous scientific questions related to synthesis, chemistry and properties of this interesting family of new materials. Novel synthesis approaches including high-pressure methods in combination with theoretical calculations offer a chance to find correct pressure/temperature conditions to realize the synthesis of the exemplarily mentioned and calculated nitrides^17^.

In the present study, we report on a discovery of a ternary nitride formed between Si_3_N_4_ and Ti_3_N_4_ synthesized under high-pressure/temperature conditions, namely at pressures of 15–20 GPa and temperatures of 1800–2000 °C in a large volume press. While the binary nitrides crystallize in different structure types (spinel-type for Si_3_N_4_ ^1^ and Th_3_P_4_-type for Ti_3_N_4_ ^18^), the formed ternary nitride reveals a spinel-type structure and it is, therefore, considered that Si in γ-Si_3_N_4_ is substituted by Ti. In addition, the ternary (Si,Ti)_3_N_4_ crystals are nano-sized and are embedded in γ-Si_3_N_4_ single crystals.

## Results

### Single-source-precursor synthesis

**Figure 2:**
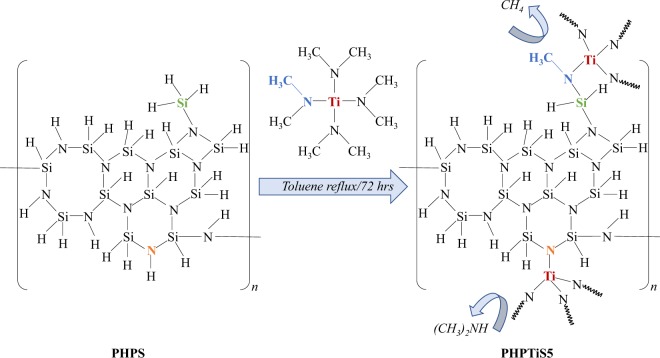
Synthesis procedure for the preparation of the SiTiN single-source precursor (SSP) according to reference^19^.

High-pressure synthesis of inorganic metal nitrides requires appropriate precursor compounds, which contain all the elements of the targeted material. In our study we first synthesized a single-source precursor (SSP) by the reaction of perhydropolysilazane (PHPS) with tetrakis(dimethylamido)titanium (TDMAT) according to the scheme given in Fig. 2 ^19,20^. For this reaction, we  choose a Si:Ti atomic ratio of 5.

**Figure 3:**
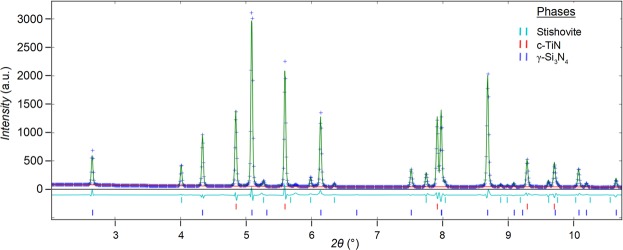
X-ray powder diffraction of SiTiN-run HH112 (λ = 0.207109 Å) and Rietveld refinement.

**Figure 4:**
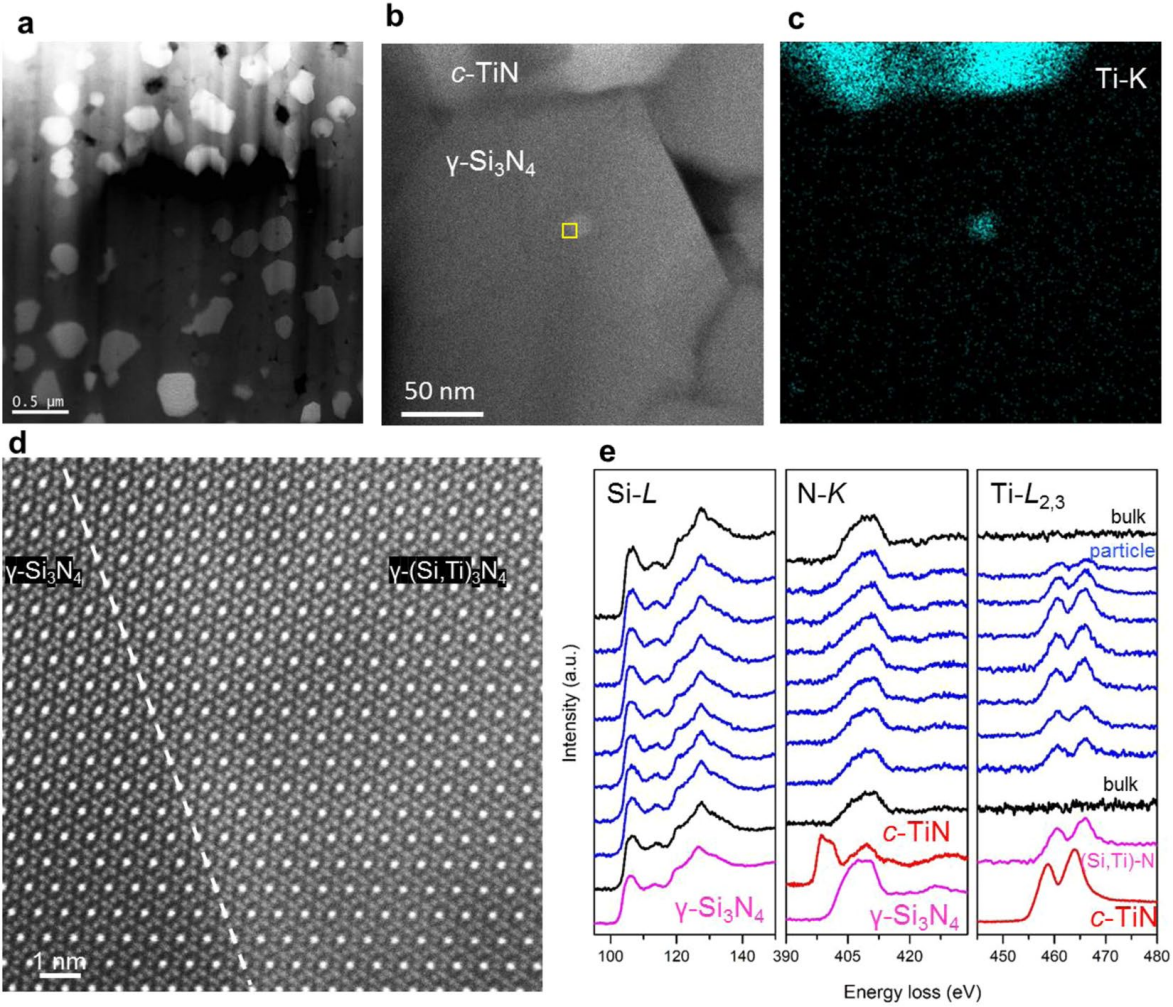
(**a**) Low-magnified ADF-STEM image, (**b**) ADF-STEM image obtained from a single grain, (**c**) Ti-K edge STEM-EDX mapping obtained from (**b**). (**d**) Atomic-resolution ADF-STEM image obtained from the yellow-framed section shown in (**b**).at the interface between γ-Si_3_N_4_ and ternary (Si,Ti)_3_N_4_ particle (**e**) EELS profiles of Si-*L*, N-*K* and Ti-*L*_2,3_ edges across the ternary (Si,Ti)_3_N_4_ nano-particle, where the black, blue, pink and red-colored profiles correspond to the bulk material, the nano-particle, γ-Si_3_N_4_ and SiTiN, and *c*-TiN, respectively.

**Figure 5:**
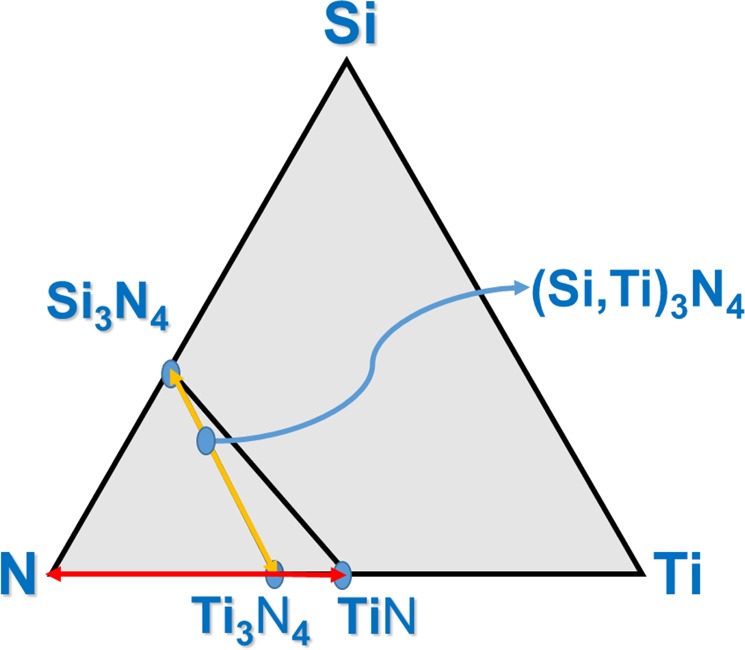
SiTiN ternary composition diagram with the known binary subsystems and the novel ternary SiTiN phase located on the tie line between γ-Si_3_N_4_ and Ti_3_N_4_.

**Figure 6:**
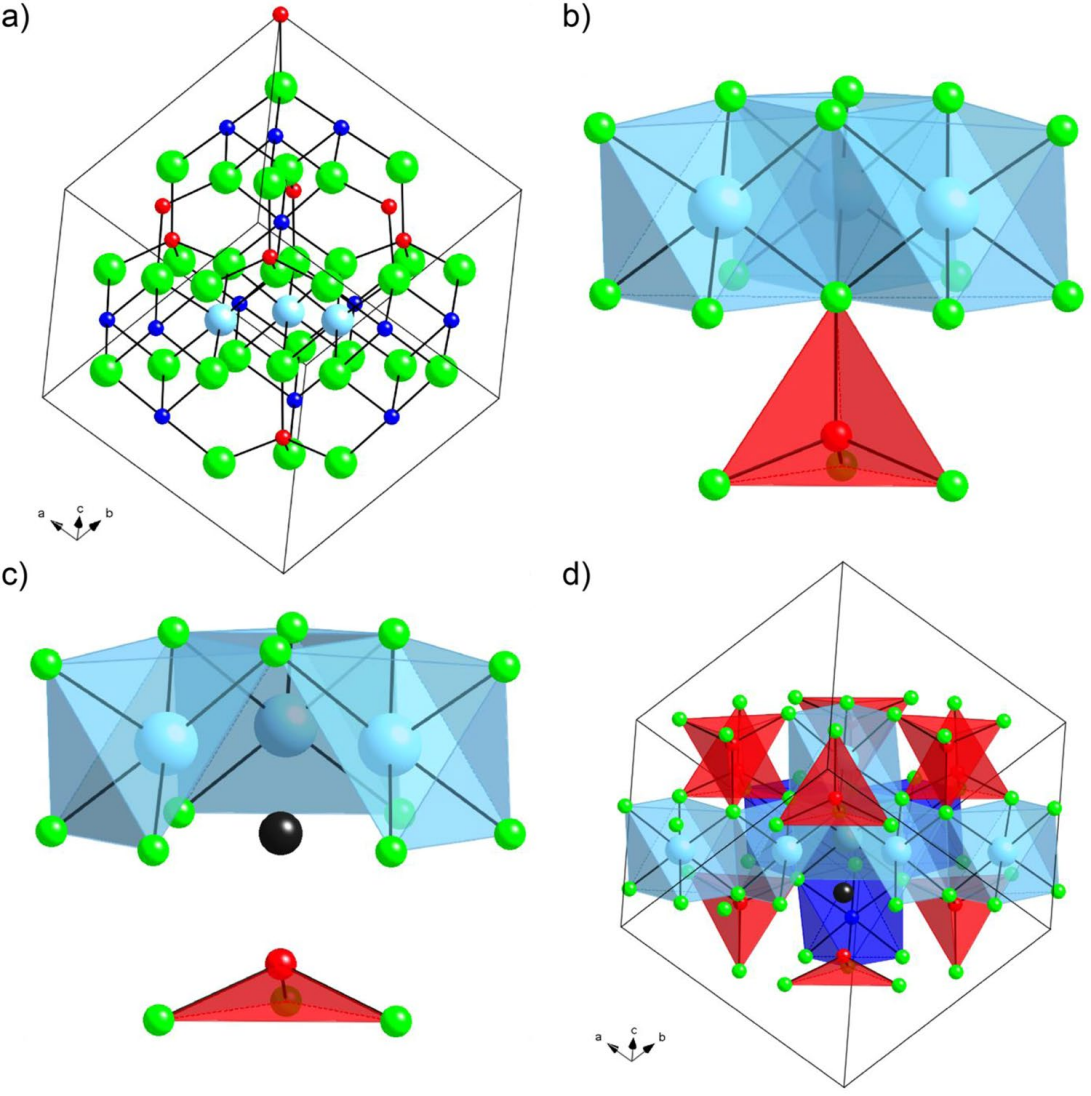
(**a**) Lowest-enthalpy structure of Si_21_Ti_3_N_32_ and (**b**) its arrangement of fused TiN_6_-cluster. (**c**) Local environment of vacancy site in Si_21_Ti_3_N_31_ exhibiting TiN_5_V-octahedra (**d**) Lowest-enthalpy structure of Si_18_Ti_6_N_31_ highlighting the chain of edge-sharing TiN_6_-/TiN_5_V-octahedra. Green spheres represent N, red and dark blue spheres tetrahedral and octahedral Si, respectively, and light blue (cyan) spheres Ti.

The obtained SSP formed via the reaction of N-H- (dimethylamine release) and Si-H- (methane release) bonds from PHPS with the ligands of TDMAT is denoted as polytitanosilazane (PHPTiS5) and the following composition was obtained by chemical analysis: 38.4 wt% Si, 17.2 wt% Ti, 7.3 wt% H, 16.0 wt% N, 21.1 wt% C and 0.9 wt% O. By neglecting the oxygen contamination, a formal composition is derived from the analytical values: Si_1.0_Ti_0.25_C_1.2_N_0.8_H_5.2_.

The SSP is finally converted to an amorphous/nanocrystalline SiTiN ceramic (labelled as PHPTiS5_1000) in ammonia atmosphere at 1000 °C with a ceramic yield of 73.25 wt%. Along with the amorphous background, the X-ray powder pattern of the synthesized SiTiN shows nanocrystalline features, which fit to the rock salt phase of TiN with a ~4.3 Å and a crystallite size of 2–3 nm. A quantitative elemental analysis of the SiTiN material revealed a composition of Si – 44.2 wt%; Ti – 18.9 wt%; N – 33.1 wt%; C – 0.02 wt%; O – 3.8 wt%. The values are used to calculate a formal composition of the synthesized powder by neglecting the carbon contamination: Si_1.0_Ti_0.2_N_1.5_O_0.15_ that represents the presence of 0.3 Si_3_N_4_ + 0.2 TiN + 0.07 SiO_2_ of the respective thermodynamically phase compositions. Thus, the Si:Ti ratio in the ammonolyzed and pyrolyzed material amounts ca. 5 and reflects that of the molar ratio used for the synthesis of the SSP.

### High-pressure synthesis

**Figure 7:**
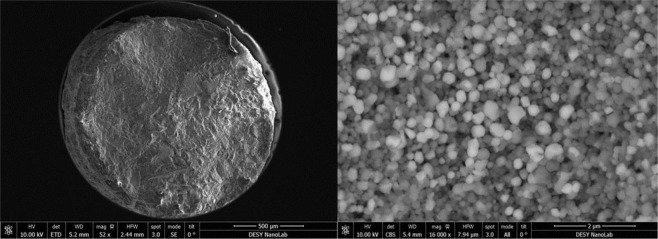
SEM micrographs (run# HH112) showing (left) an overview of the sample pellet after HP-HT and (right) a fracture surface with spinel-type γ-Si_3_N_4_ and rock salt-type c-TiN crystals.

In the second step, the acquired SiTiN powder (PHPTiS5_1000 sample) is subjected to a high-pressure/high-temperature treatment. The high-pressure experiments were performed in a large volume press under pressures between 16 and 20 GPa and temperatures between 1800 and 2000 °C. After pressure release, a compact and sintered body is isolated from the reaction chamber. A SEM image of a fracture surface of the sample exhibits a composite microstructure with dark and bright contrasted nanocrystals (see Fig. 7). X-ray diffraction analysis of the powdered sample revealed the presence of spinel-type γ-Si_3_N_4_ and rocksalt-type *c*-TiN as well as residual stishovite SiO_2_ (Fig. 3).

The fraction of the crystalline phases was obtained by Rietveld refinement of the XRD data. The fractions were 72.6 wt% γ-Si_3_N_4_, 20.2 wt% of c-TiN and 7.1 wt% Stichovite (SiO_2_) together with <0.1 wt% Pt from the capsule material of the high-pressure experiment. The Si:Ti ratio thus remained ca. 5, and the bulk sample composition of the SiTiN starting material therefore did not significantly change during experimental high-pressure/temperature treatment.

### Structural analysis via XRD and electron microscopy

The average sizes of the synthesized γ-Si_3_N_4_ and *c*-TiN were derived from the peak widths of the XRD pattern, and found both 260 ± 90 nm. Since these grain sizes are too small for chemical composition analysis by SEM-EDX, the samples were observed using ADF-STEM. As shown in Fig. 4(a), the sample consisted of two phases with different brightnesses. Since the brightness increases with increasing average atomic number, the brighter contrast grains should contain heavier elements, i.e. Ti. It is therefore concluded that the bright- and dark-contrast grains are c-TiN and γ-Si_3_N_4_, respectively. This evaluation was also confirmed by electron diffraction and EDX mapping (Fig. 4c) of this region.

On the close inspection of the grains, we found a third type of nanocrystals inside of the γ-Si_3_N_4_ grains that can be seen as a brighter dot contrast in the ADF-STEM image of Fig. 4(b), suggesting that the nanocrystal should contain Ti atoms. The embedded nanocrystals are around 10–20 nm in size and occur in the majority of the silicon nitride host crystals. The distribution of nanoparticles in γ-Si_3_N_4_ grain can be found in the low-magnification of annular bright-field (ABF) STEM image (see Supplementary Note 1). To directly confirm the validity of Ti-concentration at the nano-particle, we performed STEM-EDX mapping and the map of Ti-K edge is shown in Fig. 4(c). On the basis of the STEM-EDX mapping, we elucidate that the nanocrystal contains Si, N and Ti atoms, and moreover the concentration of Si atoms is smaller than that in the vicinity of γ-Si_3_N_4_. Thus, Ti atoms are considered to be in the substitutional form at Si atomic sites. It is important to note that no significant oxygen is found in the nanocrystals. Figure 4(d) shows an atomic-resolution ADF-STEM image viewed along the [110] orientation of γ-Si_3_N_4_, including the interface between the nanocrystal and γ-Si_3_N_4_. As indicated by the dotted line in Fig. 4(d), the nanocrystal (right-side) shows slightly brighter Z-contrast than that in the bulk of γ-Si_3_N_4_, suggesting that the nanocrystal contains Ti atoms but the structure retains spinel-type with the coherent interface between the host γ-Si_3_N_4_ and the nanocrystal: the discovery of the ternary (Si,Ti)_3_N_4_. We note that although the boundary between the nanoparticle and γ-Si_3_N_4_ may be difficult to realize from the weak Z-contrast difference, it is most evident in the Z-contrast intensity profile across the grain boundary and the simultaneously recorded ABF-STEM image (see Supplementary Note 2). For further investigation, we performed EELS analysis across the nanocrystal as shown in Fig. 4(e), where the blue-, black- and red-colored profiles are related to the nanocrystal, γ-Si_3_N_4_, and c-TiN, respectively. The pink colored profiles are obtained from the bulk of γ-Si_3_N_4_ and the nanoparticle (average for Ti-*L*_2,3_ edge). The Si-*L*, and N-*K* edges of the nanocrystal are basically similar to those in γ-Si_3_N_4_, which is well compatible with the observed atomic structure. The Ti-*L*_2,3_ edges are localized within the nanocrystal, and the peak positions of the Ti-*L*_2,3_ edge show slight upward shift compared with that in c-TiN, suggesting that Ti occurs in two oxidation states in the nanocrystal, namely Ti^3+^ and Ti^4+^. In spinel-type structure, there are two atomic sites for cations, namely octahedral and tetrahedral coordination, and the Ti oxidation states for the sites have to be considered as Ti^3+^ and Ti^4+^. For the compensation of the total charge, the nanocrystal requires N vacancies, and we conclude that the formed ternary spinel structure has to be described as (Si^4+^_1−x_ Ti^4+^_x-δ_Ti^3+^_δ_)_3_N_4-δ_, where we could not determine the Ti content (x) because the experimental spectroscopic information (EDX and EELS) is in 2D projection (the nanocrystal is embedded in γ-Si_3_N_4_ grain). However, from EELS we can roughly estimate the following ratio Ti^4+^:Ti^3+^ = (x − δ): δ ~ 0.7: 0.3, which leads to δ ~ 0.3x.

### Thermodynamic considerations

The composition of the synthesized ternary silicon titanium nitride solid solution is located on the tie line between the binary subsystems γ-Si_3_N_4_ and Ti_3_N_4_ as illustrated in the isothermal section of the phase diagram Fig. 5.

Accordingly, under the applied high-pressure/high-temperature conditions, amorphous SiTiN produced three phases, namely submicron-sized c-TiN with rocksalt-type structure and γ-Si_3_N_4_ ^1^ grains with nano-sized inclusions of a ternary γ-(Si,Ti)_3_N_4_ phase, both with spinel-type structure. With respect to the phase diagram we may hypothesize that the ternary γ-(Si,Ti)_3_N_4_ phase partitions first into γ-Si_3_N_4_ and Ti_3_N_4_ (along the yellow colored tie line in Fig. 5). However, Ti_3_N_4_ is thermodynamically unstable under our experimental conditions of 15–20 GPa and 1800–2000 °C^21^. Thus, Ti_3_N_4_ readily decomposes into the thermodynamically stable phases c-TiN and gaseous N_2_ along the red colored tie line of the binary Ti-N subsystems presented in Fig. 5 and according to the following reaction equation:

$$3\,{{\rm{Ti}}}_{3}{{\rm{N}}}_{4}\to 9\,{\rm{TiN}}+1.5\,{{\rm{N}}}_{2}$$

Moreover, crystalline Ti_3_N_4_ cannot be recovered at ambient pressure^18,21^, while the high-pressure phase of silicon-nitride shows an enormous metastability even at ambient pressure and can be heated up to 1400 °C without any phase transformation^22^. Finally, once TiN is formed from the ternary γ-SiTiN phase, the resulting γ-Si_3_N_4_/c-TiN composite is thermodynamically stable at P > 10 GPa and metastable under ambient pressure.

### Computation of Ti site preference and structure search

Guided by the experimental data we started exploring spinel-type SiTiN. A first task was to evaluate site preference of Ti^4+^ substituting for Si^4+^ in γ-Si_3_N_4_. We chose to work in the conventional unit cell of the spinel structure comprising 56 atoms. In a structure model of Si_23_TiN_32_ at ambient pressure, Ti prefers substitution of the octahedral site over a tetrahedral site. The energy difference we compute, however, is very small – only 0.01 eV. Increasing pressure increases slightly the preference for Ti to occupy the octahedral site to 0.07 eV at 15 GPa.

In a next step, we computed substitution of three Si by Ti simultaneously, still keeping Ti^4+^. Thus, within the conventional unit cell we considered Si_21_Ti_3_N_32_ and performed a structure search over all possible combinations of three Ti on cation sites. The model with the lowest enthalpy that emerged displays three Ti in octahedral sites coordinating to the same N (see Fig. 6a,b). The small cluster formed by three fused TiN_6_-octahedra resembles a fragment of the rock-salt TiN structure, albeit in this case with Ti^4+^ rather than Ti^3+^ as in TiN. The next lowest enthalpy model, still containing only Ti^4+^ in octahedral sites, is already 0.19 eV higher in energy (for the full 56 atom unit cell). The first model comprising Ti in a tetrahedral site comes out 0.28 eV higher in enthalpy than the ground state model of Si_21_Ti_3_N_32_. Interestingly, the model of Si_21_Ti_3_N_32_ with highest enthalpy (about 3 eV higher than the ground state model) also comprises all Ti in octahedral sites, but arranged with maximum distance from each other.

Introducing an anion vacancy does not change the picture of preferred cation arrangements in structures. Note that in models of Si_21_Ti_3_N_31_ all three Ti are (formally) Ti^3+^. Again, we found that Ti prefers octahedral sites and tends to cluster. With the vacancy in the model, the lowest enthalpy structure displays all Ti adjacent to the defect as **Ti**N_5_V-octahedra (see Fig. 6c). Alternative arrangements are now a bit closer in energy, the second lowest-enthalpy structure is only 0.12 eV higher than the lowest-enthalpy model. Among all combinations, the highest enthalpy structure is still one in which all Ti are octahedral, but maximally dispersed in the structure.

We finally expanded our search to Si_18_Ti_6_N_31_. This model displays half of Ti as Ti^4+^ and the other half as Ti^3+^. We limited our combinatorial search on Ti in octahedral sites, and computed only a few models explicitly considering tetrahedral Ti as well. The lowest enthalpy configuration of Si_18_Ti_6_N_31_ then shows only octahedral Ti, with three Ti in octahedral sites adjacent to the vacancy and the other Ti in different octahedral sites. Within the unit cell we considered, there now appears a chain of connected **Ti**N_6_-**Ti**N_5_V-octahedra sharing edges along the $[1\bar{1}0]$ direction. Alternatives with more dispersed arrangements are less favorable in energy. This indicates once again the preference of Ti to cluster (see Fig. 6d).

### Enthalpy of formation

To compute enthalpies of formation, ΔH_f_, of the ternary SiTiN compounds we use γ-Si_3_N_4_, TiN (for Ti^3+^), and spinel-type Ti_3_N_4_ as reference models. Note that a spinel-type Ti_3_N_4_ is not known, and only a Th_3_P_4_-type Ti_3_N_4_ has been synthesized at 72 GPa ^18^. The lowest energy structure of Ti_3_N_4_ comprising Ti^4+^ is predicted to adopt a monoclinic structure, as earlier studies have shown^23,24^.

Despite the fact that spinel-type Ti_3_N_4_ is not the ground state of Ti_3_N_4_, we nevertheless find only positive values for Δ*H*_f_ of spinel-type SiTiN. This implies that its decomposition into binary compounds is favored by enthalpy. An anion vacancy and presence of some Ti^3+^ only increases the trend and so does pressure. With the Δ*H*_f_ spinel-type SiTiN being positive, we turn to estimate the entropy of mixing, ∆*S*_mix_, arising from distributing Ti among the cation sites and a vacancy among anion sites^25,26^. Neglecting site preferences, which will only reduce our estimate of ∆*S*_mix_, we estimate an upper bound of ∆*S*_mix_ of 749, 1160, 1430 J/(K·mol), for Si_21_Ti_3_N_32_, Si_21_Ti_3_N_31_, and Si_18_Ti_6_N_31_, respectively. Since less vacancies are present than Ti cations, the cation mixing contributes to the total entropy of mixing more than the anion mixing. With ∆*G* = ∆*H*_f_ − *T*∆*S*_mix_, we estimate that it would require temperatures greater than 5000 K to “stabilize” Si_21_Ti_3_N_31_ and Si_18_Ti_6_N_31_ against decomposition into the ternary system γ-Si_3_N_4_, TiN, and spinel-type Ti_3_N_4_. We also considered a superstructure of *α*-Si_3_N_4_ and substituted Si partially by Ti. This structure, thus, comprises tetrahedrally coordinated Ti^4+^ and may serve as a model for the precursor used during synthesis of spinel-type SiTiN. Not surprisingly, the lowest enthalpy structure of such a α-Si_21_Ti_3_N_32_ displays all Ti clustered and bonding to the same N. Moreover, we find that incorporation of Ti slightly reduces the pressure of the α−γ transition by 0.4 GPa, if we compare our models of α-Si_21_Ti_3_N_32_ and γ-Si_21_Ti_3_N_32_ discussed above relative to pure Si_3_N_4_ structures.

In summary, the occurrence of spinel-type SiTiN cannot be attributed to thermodynamical stability. A spinel-type (Si,Ti)_3_N_4_ solid solution is less favorable than assemblages of separated phases of Si_3_N_4_, TiN, and Ti_3_N_4_. However, the spinel-type (Si,Ti)_3_N_4_ solid solution is more favorable than the precursor system with tetrahedral Ti^4+^ mixed into a silicon nitride at 15 GPa. Therefore, formation of spinel-type (Si,Ti)_3_N_4_ should be due to the sluggish kinetics, because formation of separate phases from the uniform precursor is a slower process than formation of a single phase of the spinel-type (Si,Ti)_3_N_4_ solid solution.

### Ti oxidation state

An interesting question is related to the different charge states identified for Ti in spinel-type (Si,Ti)_3_N_4_. The compound comprises both Ti^3+^ and Ti^4+^. For a characterization of charges, we computed the lowest energy configuration of Si_21_Ti_3_N_32_, Si_21_Ti_3_N_31_, and Si_18_Ti_6_N_31_, and used Bader charge analysis^27,28^. In Si_21_Ti_3_N_32_, which displays Ti^4+^, we find 2.8 e^–^ around the Ti according to the Bader charge analysis. On the other hand, Ti^3+^ in Si_21_Ti_3_N_31_ exhibits higher Bader charges of 3.2 e^−^. In Si_18_Ti_6_N_31_, which formally has equal proportions of Ti^3+^ and Ti^4+^, we indeed find half of the Ti with Bader charges of 2.0 e^−^, while the other half displays Bader charges between 2.6 and 2.9 e^−^. This allows us to identify the two cohorts as Ti^4+^ and Ti^3+^, respectively. Noteworthy is that Ti^3+^ appears adjacent to the vacancy as **Ti**N_5_V-octahedra, while Ti^4+^ appears within **Ti**N_6_-octahedra.

### Elastic properties

We also computed elastic constants for all compounds and estimate elastic moduli (B, G) as well as Vickers hardness H_V_, see Table 1. The γ-Si_3_N_4_ is a notably hard material^29^, but adding Ti reduces elastic properties and decrease hardness. The estimated hardness of Si_18_Ti_6_N_31_ comes out to be close of half of that of γ-Si_3_N_4_.

**Table 1:** Bulk modulus B, shear modulus G, and Vickers hardness H_V_ of Si_3_N_4_ and SiTiN compounds with the spinel structure type at ambient pressure using SCAN functional.

	B (GPa)	G (GPa)	H_V_ (GPa)
Si_3_N_4_	333	283	42
Si_21_Ti_3_N_32_	327	248	33
Si_21_Ti_3_N_31_	308	234	32
Si_18_Ti_6_N_31_	292	204	26

### Electronic properties

We computed the band gap of γ-Si_3_N_4_ to 4.95 eV using the screened hybrid HSE-functional^30^. This aligns with previous calculations and available experimental data^31^. Replacing Si^4+^ by Ti^4+^ introduces localized unfilled d-states below the conduction band, ultimately decreasing the width of the band gap. Introducing a single nitrogen vacancy creates an odd number of Ti^3+^ and results in a partially filled band in the middle of the gap.

## Discussion

In summary, an amorphous SiTiN labelled PHPTiS5_1000 compound was synthesized by the reaction of perhydropolysilazane with tetrakis(dimethylamido)titanium and subsequent heat-treatment in ammonia at 1000 °C. The SiTiN PHPTiS5_1000 was exposed to 15–20 GPa pressure and 1800–2000 °C in a large volume press to form a high-pressure γ-Si_3_N_4_/c-TiN composite material for the first time. The special feature of the obtained composite is due to the discovery of an additional nanocrystalline ternary γ-(Si,Ti)_3_N_4_ phase with spinel-type structure embedded within the majority of the submicron γ-Si_3_N_4_ crystals. The γ-(Si,Ti)_3_N_4_ phase was analyzed in detail by TEM and EELS and the results clearly showed the presence of two types of Ti atoms, namely Ti^3+^ and Ti^4+^, which in turn requires the formation of vacancies in the anion lattice. Therefore, the general formula of the discovered ternary spinel-type SiTiN has to be described as (Si^4+^_1−x_ Ti^4+^_x-δ_Ti^3+^_δ_)_3_N_4-δ_. According to theoretical calculations, Ti^3+^ appears adjacent to the vacancy as **Ti**N_5_V-octahedra, while Ti^4+^ appears within **Ti**N_6_-octahedra. Moreover, all of the ternary SiTiN compounds have been calculated to be thermodynamically unstable with respect to the binary phases, which is in accordance with the experimental result, that finally a binary γ-Si_3_N_4_/c-TiN high-pressure composite is formed.

In consequence, it has to be noted that the synthesis of the ternary spinel-type SiTiN phase is kinetically controlled and requires an appropriate precursor material (SSP) with Ti^4+^ atoms in tetrahedral coordination together with tetrahedrally coordinated Si in amorphous silicon nitride. A direct reaction of Si_3_N_4_ with TiN to form a ternary high-pressure SiTiN phase is thermodynamically impossible and has not been observed yet experimentally. The present work encourages to study other SSP-derived Si-based systems containing transition metal atoms other than Ti to form innovative high-pressure composite materials and novel inorganic ternary metal nitrides.

## Methods

### Single-source-precursor synthesis

A titanium-modified perhydropolysilazane (PHPS) as single source precursor (SSP) was synthesized by a reaction between PHPS and tetrakis(dimethylamido)titanium (TDMAT) with a molar Si:Ti ratio 5:1 (labelled as PHPTiS5) according to our previous reports^19,20^.

The manipulation of the PHPTiS5 sample was made in an argon-filled glove box (Jacomex, Campus-type; O_2_ and H_2_O concentrations kept at 0.1 ppm and 0.8 ppm, respectively) to be ground in an agate mortar and then placed in alumina boats. Alumina boats containing the polymers were introduced in tubes under protective atmospheres (argon) to be transferred under argon flow into a silica tube inserted in a horizontal furnace [THERMOCONCEPT OS50/450/12]. After evacuation of the furnace under dynamic vacuum (0.1 mbar), the tube was refilled with ammonia up to atmospheric pressure and a continuous flow of ammonia was maintained through the tube. Subsequently, the samples were pyrolyzed at a heating rate of 5 °C min^−1^ to 1000 °C (dwell time for 2 h) in order to produce the SiTiN precursor (labelled as PHPTiS5_1000). After cooling at a rate of 2 °C min^−1^ under nitrogen flow, the PHPTiS5_1000 was used as a starting material for the subsequent high pressure synthesis.

The silicon and titanium contents of the synthesized PHPTiS5_1000 sample powders were measured by inductively coupled plasma/optical emission spectroscopy (ICP/OES) [Optima 8300 optical emission spectrometer Perkin Elmer, USA]. The carbon and the oxygen, nitrogen and hydrogen contents of the powders were determined by combustion elemental analysis [Carbon (EMIA-321V), O/N/H analyzer (EMGA-830) Horiba, Japan].

The phase composition of PHPTiS5_1000 sample was determined by powder X-ray diffraction (XRD) with a Bruker AXS D8 Discover and Cu*K*_α_ radiation. The scans were performed in the range of 2θ ∈ 〈15°; 90°〉 with a step of 0.015° and an exposure time of 0.7 s. The diffraction patterns were analyzed using the Diffrac + EVA software with the JCPDS-ICDD database.

### High-pressure synthesis

High-pressure high-temperature experiments were performed in a Hall-type six-ram LVP (mavo press LPQ6 1500–100; Max Voggenreiter GmbH, Germany) installed at the P61B beamline at DESY, Hamburg^32^. The samples were synthesized in a 14/7 or 10/4 assembly of eight tungsten carbide cubes (32 mm Fujilloy TF08) at the target pressures. A Cr_2_O_3_-doped MgO octahedron was used as the pressure-transmitting medium. The starting material was cold pressed into pellet form (h = 1.6 mm, ø =1.9 mm) using a platinum foil before placing in the MgO tube. The octahedral assembly was compressed to a pressure of 16 and 20 GPa and, at pressure, heated to a chosen temperature in the range of 1800–2000 °C and held for 30 min. Pressure was calibrated at room temperature using the semiconductor to metal transition of GaP at 22 GPa^33^. Sample temperatures were estimated using power-temperature relations calibrated in a separate run using a W5%Re/W26%Re thermocouple (C-type). Overview of experimental conditions with the corresponding run numbers are given in Table 2.

**Table 2:** Overview of HP-HT experiments with run number, assembly, pressure, temperature, heating duration and capsule materials.

Run No	Sample	Assembly	Pressure (GPa)	Temperature (°C)	Heating duration (min)	Capsule material
HH113	SiTiN-PHPTiS5_1000	14/7	16	1800	30	Pt
HH109	SiTiN-PHPTiS5_1000	10/4	20	1800	30	Pt
HH112	SiTiN-PHPTiS5_1000	10/4	20	2000	20	Pt

After recovery, each sample was found dense and fine-grained in appearance (see Fig. 7).

XRD of crushed samples was obtained using synchrotron radiation at the high-resolution powder diffraction beamline P02.1 of PETRA-III, DESY, Hamburg. The XRD pattern was quantitatively analyzed by Rietveld refinement with the program GSAS-II^34^.

### Electron microscopy

For the preparation of electron transparent thin specimen, the synthesized bulk sample was processed by focused ion beam (Helios G4, Thermo Fisher Scientific) and subsequently, to clean the specimen, Ar ion beam milling was performed at 0.5 kV in the final step. For the atomic and electronic structure analysis, we used an aberration-corrected STEM (JEM ARM-200CF, JEOL Ltd.), equipped with an annular dark-field (ADF) and annular bright-field (ABF) detectors, EEL spectrometer (Enfinium, Gatan Inc.) and dual electron dispersive X-ray (EDX) spectrometers. The electron microscope was operated at 200 kV. For atomic-scale imaging and spectroscopy, the illumination semi-angle was 24 mrad, and the ABF and ADF detectors span from 12 to 24 and 64 to 200 mrad, respectively, where a typical beam current was 20 pA.

### Computational method

All calculations were performed using density functional theory (DFT) as implemented in the Vienna *ab initio* simulation package (VASP)^35–38^. For correlation and exchange we use the Strongly Conserved and Appropriately Normed (SCAN) functional together with the projector-augmented-wave (PAW) method^39–41^. Reported results were obtained using a plane wave cut-off energy of 500 eV. We sample the Brillouin zone of the conventional unit cell and all derived structures using a 2 × 2 × 2 k-point mesh. With parameters reported above, enthalpy differences between structures are converged to better than 0.005 eV per conventional unit cell.

Different models of spinel-type Si_21_Ti_3_N_32_ and Si_21_Ti_3_N_31_ were investigated by testing all the possible combinations of Ti at Si octahedral and tetrahedral sites. We first identified lowest enthalpy structures by static calculations without further relaxation. Structures with low enthalpies were further optimized until forces converged to 0.01 eV/Å and residual stresses are below 0.01 GPa. It turned out that this optimization does not change the ranking of the models to a significant extent. Lowest enthalpy models of each composition were further optimized (forces converged to 5 meV/Å) and used for subsequent calculations. The lowest enthalpy model of Si_18_Ti_6_N_31_ attained monoclinic structure (SpGr. *C*1*m*1 (8); a = 7.905 Å, b = 7.905 Å, c = 7.923 Å, α = 89.94°, β = 89.94°, γ = 89.68°), but displayed only small distortion away from a pseudo-cubic cell. Substituting Ti for Si increases the cell volume relative to spinel-Si_3_N_4_ even in models with a vacancy.

## Supplementary information

Supplementary information.
