# Supplementary material for: Discovery of Ternary Silicon Titanium Nitride with Spinel-Type Structure
Source: Sci Rep. 2020 Apr 30;10:7372. doi: 10.1038/s41598-020-64101-5 (PMC7193582; doi:10.1038/s41598-020-64101-5)
Supplement: Supplementary file 1 — Supplementary information. [file 41598_2020_64101_MOESM1_ESM.pdf]

Supplementary Information

Discovery of Ternary Silicon Titanium Nitride with Spinel-Type Structure

Shrikant Bhat[1*], Abhijeet Lale[2], Samuel Bernard[2], Wei Zhang[3], Ryo Ishikawa[3,4], Shariq Haseen[5], Peter Kroll[5], Leonore Wiehl[7], Robert Farla[1], Tomoo Katsura[6], Yuichi Ikuhara[3] and Ralf Riedel[7]

[1] Deutsches Elektronen-Synchrotron DESY, D-22607 Hamburg, Germany

[2] University of Limoges, CNRS, IRCER UMR 7315, F-87000, Limoges, France

[3] Institute of Engineering Innovation, University of Tokyo, Bunkyo, Tokyo, 113-8656, Japan

[4] Japan Science and Technology Agency, PRESTO, Kawaguchi, Saitama 332-0012, Japan

[5] Department of Chemistry and Biochemistry, University of Texas at Arlington, Arlington, Texas 76019, United States

[6] Bayerisches Geoinstitut (BGI), University of Bayreuth, 95440 Bayreuth, Germany

[7] Technische Universität Darmstadt, Fachbereich Material- und Geowissenschaften, Fachgebiet Disperse Feststoffe, Otto-Berndt-Str. 3, D-64287 Darmstadt, Germany

* Corresponding author: shrikant.bhat@desy.de

Supplementary Note 1. The statistics of new ternary nanocrystals.

We frequently observed the ternary nanocrystals in γ-Si_3N_4 grains. In the low-magnification of ADF-STEM image in Fig. 4(a), the grains of TiN show considerably strong contrast and it is difficult to find the nanocrystals. To clear the statistics on the nanocrystals, we here show bright-field (BF) STEM image of Suppl. Fig. S1 (a), where the contrast range is compressed compared with ADF-STEM image. In BF-STEM, a grain containing heavy elements basically becomes dark and therefore the dark-dot contrasts as marked by red circles correspond to our new ternary nanocrystals. To confirm the nanocrystals with dark-dot contrasts in Suppl. Fig. 1(a), we acquired ADF-STEM image obtained from the white rectangle region in (a), and the ADF-STEM image show the bright-contrast, suggesting that the nanocrystal contains heavy elements, i.e. Ti atoms. As shown in Suppl. Fig. S1(a), one can find many nanocrystals in γ-Si_3N_4 grains (not in all the grains), and therefore we conclude that the nanocrystal can be considered as a new ternary phase.

Supplementary Note 2. Grain boundary between γ-Si_3N_4 and the ternary nanocrystal.

When we observe the crystal along the low index zone axis, the dynamical effect become significant and it may lead to difficult to see the grain boundary between γ-Si_3N_4 and the ternary nanocrystal as shown in Fig. 4(d). To confirm the grain boundary of Fig. 4(d), we show the ADF intensity profile along X-X' direction, as shown in Suppl. Fig. S2(a) and (c). The ADF intensity is evidently increased across the grain boundary. For more confidence, we also show the simultaneously recorded annular bright-field (ABF) STEM image in Suppl. Fig. S2(b), where one can clearly see the contrasts difference between γ-Si_3N_4 and the ternary nanocrystal.

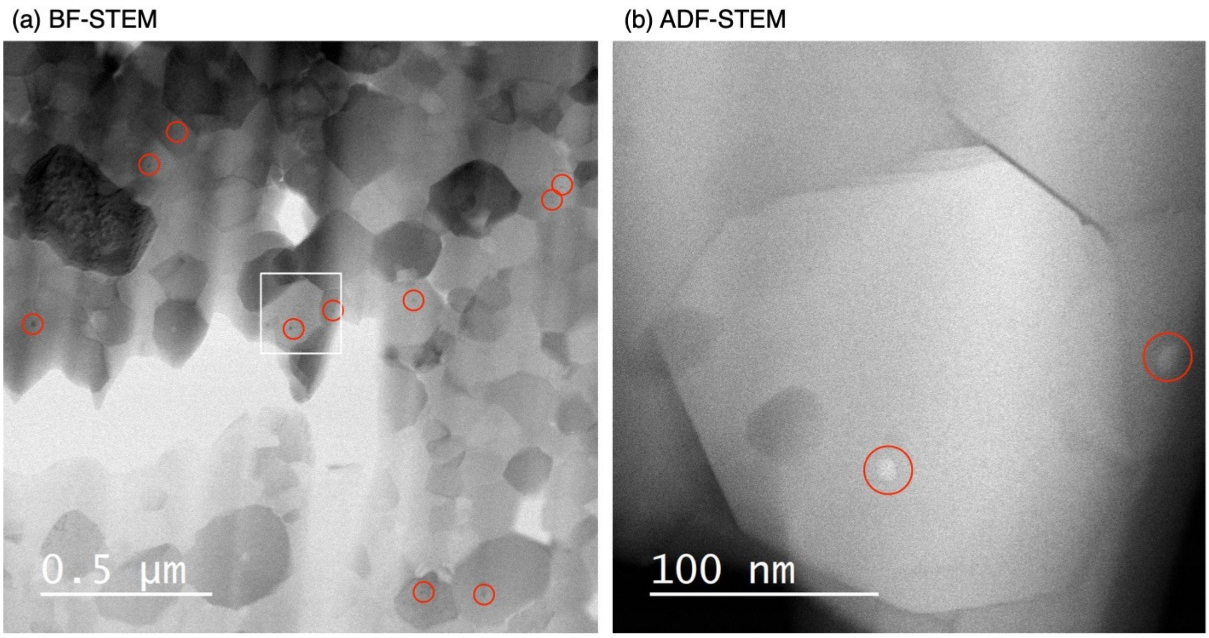

Supplementary Figure 1. (a) BF-STEM image and (b) the ADF-STEM image obtained from the white rectangle region in (a). The red circles correspond to the new ternary nanocrystals.

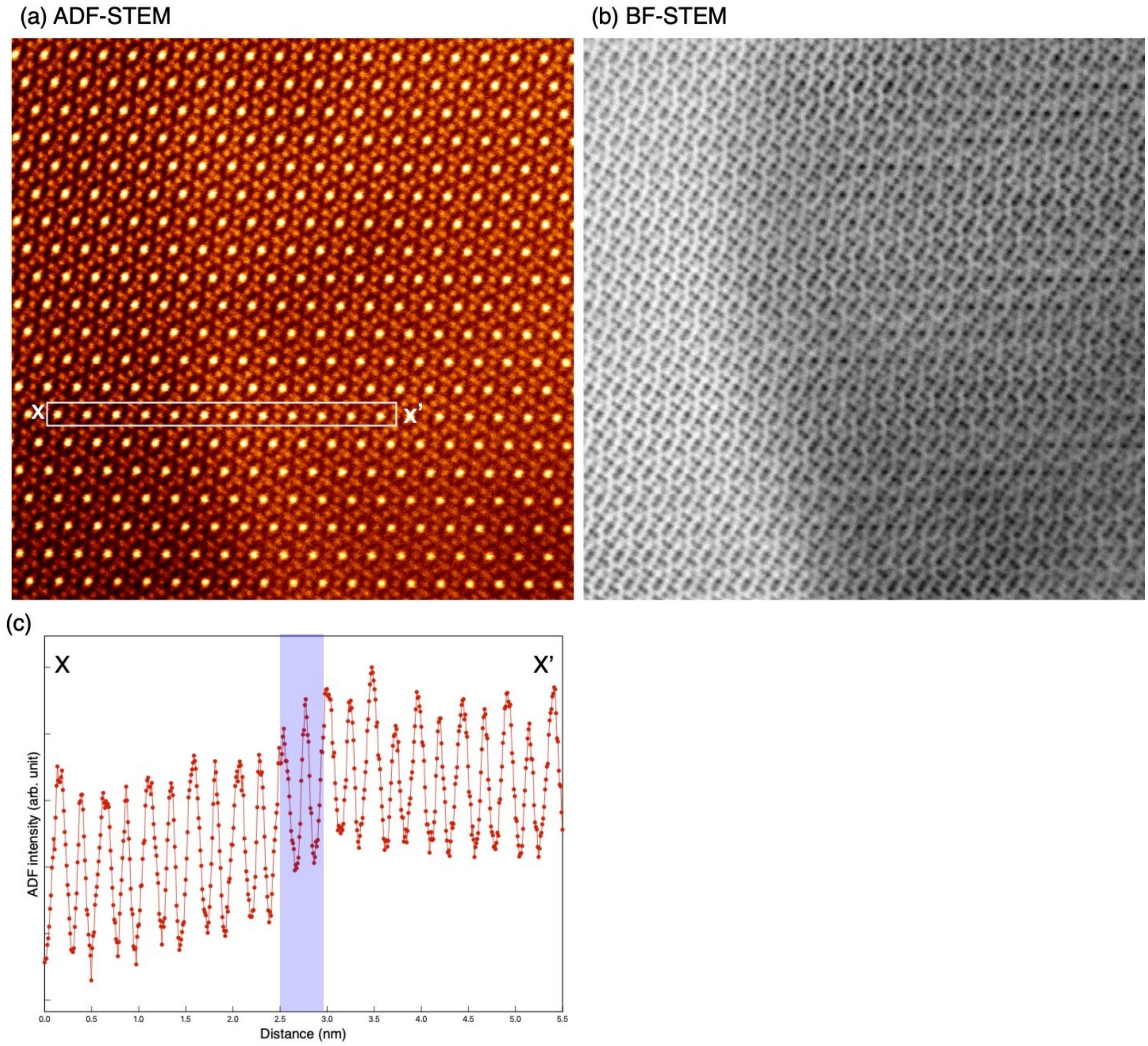

Supplementary Figure 2. The simultaneously recorded atomic-resolution (a) ADF-STEM and (b) ABF-STEM images, respectively. (c) The ADF intensity profile along X-X' direction in (a), where the blue-rectangle region correspond to the position of the grain boundary.
